# Pathophysiology and clinical management of coronavirus disease (COVID-19): a mini-review

**DOI:** 10.3389/fimmu.2023.1116131

**Published:** 2023-08-14

**Authors:** Ying Zhu, Lokesh Sharma, De Chang

**Affiliations:** ^1^ College of Pulmonary and Critical Care Medicine, 8th Medical Center of Chinese PLA General Hospital, Beijing, China; ^2^ Department of Pulmonary and Critical Care Medicine, 7th Medical Center of Chinese PLA General Hospital, Beijing, China; ^3^ Section of Pulmonary and Critical Care and Sleep Medicine, Yale University School of Medicine, New Haven, CT, United States

**Keywords:** pathophysiology, clinical management, SARS-CoV-2, COVID-19, prevention

## Abstract

An unprecedented global pandemic caused by a novel coronavirus named SARS-CoV-2 has created a severe healthcare threat and become one of the biggest challenges to human health and the global economy. As of July 2023, over 767 million confirmed cases of COVID-19 have been diagnosed, including more than 6.95 million deaths. The S protein of this novel coronavirus binds to the ACE2 receptor to enter the host cells with the help of another transmembrane protease TMPRSS2. Infected subjects that can mount an appropriate host immune response can quickly inhibit the spread of infection into the lower respiratory system and the disease may remain asymptomatic or a mild infection. The inability to mount a strong initial response can allow the virus to replicate unchecked and manifest as severe acute pneumonia or prolonged disease that may manifest as systemic disease manifested as viremia, excessive inflammation, multiple organ failure, and secondary bacterial infection among others, leading to delayed recovery, hospitalization, and even life-threatening consequences. The clinical management should be targeted to specific pathogenic mechanisms present at the specific phase of the disease. Here we summarize distinct phases of COVID-19 pathogenesis and appropriate therapeutic paradigms associated with the specific phase of COVID-19.

## 1 Introduction

The ongoing COVID-19 pandemic is entering its fourth year which began with the identification of a group of patients with unknown pneumonia in Wuhan, China in December 2019 (1). This is the third major coronavirus outbreak preceded by severe acute respiratory syndrome (SARS) and Middle East respiratory syndrome (MERS), however, both of these viruses were contained before causing the global pandemic. Since the emergence of COVID-19, a significant proportion of the human population has been infected with SARS-CoV-2, the causative virus of COVID-19 (2). The rapid spread of the virus allowed the virus to evolve quickly to become more infectious. Multiple variants of the virus named Alpha, Beta, Gamma, Delta, and Omicron, have emerged during this pandemic and repeated infections have become more common. Many of these variants have much higher infectivity compared to the original strain (3, 4). The continuous evolution of SARS-CoV-2 has caused unprecedented devastating effects on human health and the global economy. As of July 2023, more than 767 million cases of confirmed COVID-19 have been reported and the virus has claimed over 6.95 million lives (5). Beyond the acute disease and death, the COVID-19 pandemic has touched every aspect of life including economic well-being, mental health, and impaired services for other diseases leading to an increase in mortalities due to other diseases (6). Additional impacts include impaired learning in children and stress among adults leading to increased rates of suicide and self-harm (7).

Individuals infected by SARS-CoV-2 present with a wide range of disease severity ranging from asymptomatic to severe disease that leads to death. While in most of the subjects, COVID-19 manifests as a mild flu-like disease, a small but significant number of patients develop severe and often life-threatening diseases. A multitude of factors contributes to the disease severity including viral load, inflammation, and immune response equilibrium. The life-threatening condition occurs due to excessive inflammation and/or impaired viral clearance caused by aging, underlying diseases such as diabetes and hypertension, and many other unknown factors (8, 9). Beyond the acute disease, the chronic consequences of COVID-19 are being documented that include severe life-altering changes such as chronic fatigue, impaired memory, and cognitive functions, among others (10, 11).

Significant progress has been made in our understanding of the transmission mechanisms, preventive measures, vaccinations, and therapeutic approaches to treat COVID-19. Despite these advances, COVID-19 remains one of the foremost healthcare challenges with significant morbidity, mortality, and economic costs each day across the world. Further, the continuous emergence of novel strains threatens our progress regarding both preventative and therapeutic approaches as immune evasion and antiviral resistance are likely consequences of viral evolution.

The chronic sequelae of COVID-19, known as long COVID occurs in at least 10% of the infected individuals and poses a global health challenge (12, 13). More than 200 symptoms have been documented affecting multiple organ systems, with many patients experiencing multiple symptoms simultaneously, affecting their quality of life including the ability to return to work (14, 15). Unfortunately, no validated effective treatments currently exist. This review provides updated insights into the pathophysiology of COVID-19 and available therapeutic avenues to treat this deadly disease, with the aim of reducing COVID-19 incidence, hospitalization, mortality, and long COVID.

## 2 Pathophysiology

### 2.1 Interactions and entry of the SARS-CoV-2 into the cell

Confirmed COVID-19 patients with symptoms and asymptomatic carriers are the primary source of new infections (16). In addition to the respiratory droplets and contact with contaminated surfaces, infection by fecal-oral route has been speculated (17). When SARS-CoV-2 initially infects people, the viral spike (S) protein binds to the angiotensin-converting enzyme 2(ACE2) receptor, which mediates the entry of SARS-CoV-2 into host cells such as nasal, bronchial epithelial cells and pneumocytes (18). The binding affinity of the S protein of SARS-CoV-2 with ACE2 is 10-20 folds higher than that of SARS-CoV, potentially explaining the quick spread of this pandemic (19). S protein undergoes further priming by type 2 transmembrane serine protease (TMPRSS2), a cellular protease particularly present in alveolar epithelial type II cells, which promotes viral uptake and coronavirus entry. Generally, the ACE2 receptor is expressed in multiple tissue cells, including airways, cornea, esophagus, ileum, colon, liver, gallbladder, heart, kidney, and testis. TMPRSS2 expression has an even broader distribution implicating that ACE2, rather than TMPRSS2, may be a limiting and major factor for viral entry at the early stage of the infection (20, 21). Notably, ACE2 and TMPRSS2 can be targeted for drug intervention to prevent the invasion and transmission of SARS-CoV-2 in host cells (22, 23). To further explain the mechanism of viral entry into host cells, the binding of the S protein to ACE2 in the viral entry process involves several stages:

Attachment: The S protein of SARS-CoV-2 binds to the ACE2 receptor on the surface of the host cell.

Priming: The S protein is then cleaved by a host protease enzyme called TMPRSS2. This cleavage allows the S protein to undergo a conformational change, exposing a fusion peptide that facilitates the fusion of the viral membrane with the host cell membrane.

Fusion: The viral membrane fuses with the host cell membrane, allowing the viral genetic material (RNA) to enter the host cell.

Replication: Once inside the host cell, the viral RNA is used as a template to produce more viral proteins and RNA.

Assembly and Release: The newly produced viral proteins and RNA assemble into new viral particles, which are then released from the host cell to infect other cells. Overall, the binding of the SARS-CoV-2 S protein to the ACE2 receptor on host cells plays a crucial role in the viral entry process and the subsequent development of COVID-19. Understanding the molecular mechanisms underlying this process is crucial for developing effective strategies to prevent and treat COVID-19 (15–18). More recent evidence has indicated ACE2 independent entry of SARS-CoV-2 into the cells, especially in the immune cells (24). The pathological consequences of ACE2-independent entry of the SARS-CoV-2 into host cells are not completely understood. Further, it remains unclear if the extra-pulmonary manifestations such as multi-organ failure are direct consequences of viral infection to those tissues or a consequence of host inflammatory response.

### 2.2 Early stage of infection

Upon entry into cells in the upper respiratory tract, SARS-CoV-2 starts to replicate and propagate in the nose and upper airways. Although infected subjects may remain asymptomatic at this stage, they are highly infectious with a high viral load, which begins to peak around symptom onset (25, 26). Subsequently, the virus may migrate from the nasal epithelium to the upper respiratory tract via the ciliated cells in the conducting airways (18, 27). The infected individuals can shed viral particles by not only coughing or sneezing but also during their day-to-day activities such as talking, eating, and even exhaling. Pre-symptomatic transmission is considered a significant contributor to viral transmission responsible for 9.1%-62% of positive cases which vary from different literatures among different populations (25, 28, 29).

**Figure 1:**
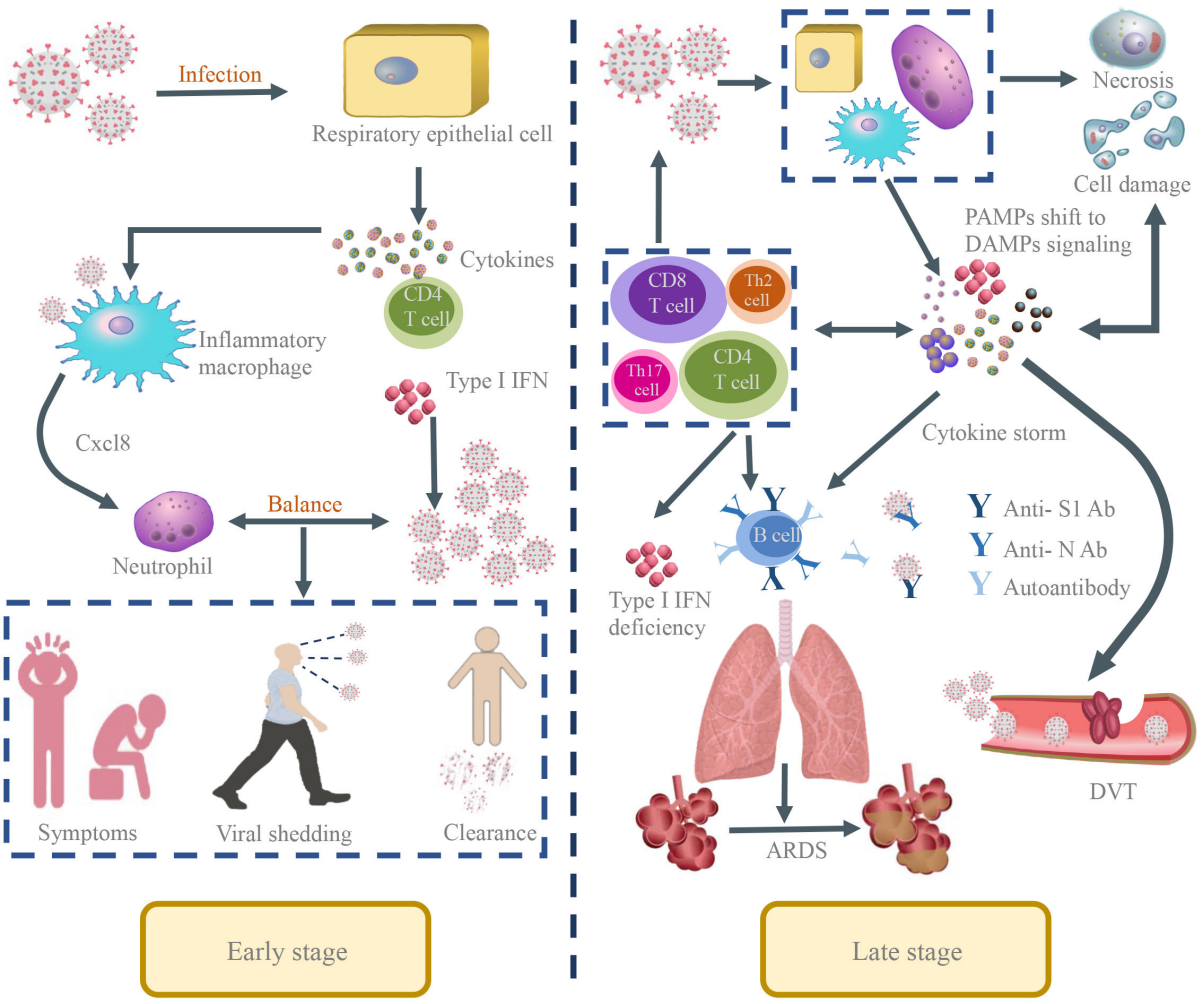
The pathological manifestations of SARS-CoV-2 infection in the human body. Early stage: In the early stage of SARS-CoV-2 infection, the respiratory epithelial cells were targets of the virus and the virus begins to replicate and spread in the nose and upper respiratory tract. The host mounts an innate response mediated by cytokines and antiviral interferons that initiates the adaptive immune response. In the early stages, infected people show the symptoms of fever, malaise, cough and sputum production and can transmit viruses to other people. If the host can mount a strong interferon-mediated response at this stage and appropriate regulation of the host immunity relative to the viral burden, viral replication would be arrested and viral clearance initiated to limit disease severity at this stage. Late stage: If the virus can’t be eradicated in a timely manner likely due to a delayed PAMP-mediated inflammatory/interferon response, the immune response would shift to a nonspecific inflammatory reaction dominated by damage-associated molecular pattern (DAMP) signaling emanating from damaged or dysfunctional virus-infected host cells. Immune cells including CD4 helper T cells, and CD8 cytotoxic T cells are sequestered in the lung tissue. The host cells undergo persistent apoptosis or necrosis or pyroptosis that may amplify the tissue damage. In addition, the inflammatory environment triggers the expression of activated tissue factor on endothelial cells, macrophages, and neutrophils, thereby enhancing activation of the coagulation cascade in the lungs, causing microcirculatory thrombi and ARDS and increasing disease severity and mortality.

The viral transmission also occurs during the symptomatic disease and may even continue after symptoms are solved (30). Infected individuals manifest the symptoms of fever, malaise, cough, and sputum production at the early stage. The host mounts an innate response that is mediated by cytokines and antiviral interferons and initiates the adaptive immune response. If the host is able to mount a strong interferon-mediated response at this stage, such as seen in children and adults, they may control the viral replication and limit the disease severity at this stage (26, 31, 32). The precise mediators of early viral clearance are not yet completely understood but given the potent antiviral activity and robust upregulation in those with a mild disease show a critical role of interferons in viral elimination (**Figure 1**) (32, 33).

### 2.3 Late stage of infection

Subjects that fail to eradicate the virus in its early stage, may progress to the clinical phase or later stage of the infection, which is manifested by COVID-19 symptoms that may vary in severity and duration (34). It is estimated that 1/5th of the infected patients progresses to the involvement of the lower respiratory tract that involves infection to the alveolar epithelial type II cells, developing severe symptoms like acute respiratory distress syndrome (ARDS), disseminated intravascular coagulation (DIC), and pulmonary embolism. The clinical phase of SARS-CoV-2 can be characterized into three distinct phases: acute or pneumonia phase, viremia phase, and lethal/recovery phase.

The acute phase is characterized by pulmonary disease that is manifested by pulmonary symptoms such as dyspnea, cough, and sputum production with imaging evidence of ground-glass opacity or consolidation in the lung. Diffuse alveolar damage, desquamation of pneumocytes, and hyaline membrane formation are observed during the development of ARDS in COVID-19 (35). The increased permeability of the lung vasculature impairs oxygen diffusion and contributes to the fatal disease. Factors contributing to lung permeability during COVID-19 are multifactorial and may include 1. Direct cytopathic effects of coronavirus in infected endothelium resulting in widespread endothelialitis (36). 2. The reduction of ACE2 activity by SARS-CoV-2 and subsequent increase in angiotensin indirectly boosting the kallikrein-bradykinin pathway which promotes vascular permeability (37). 3. Inflammatory cytokines and vasoactive mediators secreted by immune cells such as activated neutrophils induce the contraction of endothelial cells and loosen endothelial tight junctions. 4. Glycocalyx degradation and hyaluronic acid deposition in the extracellular matrix promote fluid retention (38). The increased lung vascular permeability results in impaired lung function that manifests into decreased blood oxygenation, a marker of disease severity.

The viremic phase begins when the virus enters the peripheral blood. The molecular mechanisms of viremia in COVID-19 are still poorly understood, however, ACE2 independent entry of the virus in peripheral monocytes has been shown to promote pyroptotic cell death and disease severity (24). The viremia and subsequent host response contribute to multiple systemic inflammation and multiorgan failure. The inflammatory response during severe COVID-19 is mediated by a simultaneous increase in the multiple inflammatory cytokines such as IL-1α, IL-1β, IL-6, IL-8, IL-12, IL-17), TNF- α, interferons (IFN- β, IFN- λ), MCP-1, and MIP-1 α, making it difficult to pinpoint the specific mediator of inflammatory response (39). Additionally, the early inflammatory response may help the host to limit viral replication, further complicating the role of cytokines in COVID-19. It is not surprising that therapies targeting specific cytokines such as IL-6 or TNF- α led to mixed results (40, 41).

The lethal phase is mediated by a persistent disease that is manifested both locally and systemically. The inflammatory response in the form of cytokine storm and coagulation factors is significantly elevated in severe patients compared to non-severe patients (42–44). In this phase, neutrophils, CD4 helper T cells, and CD8 cytotoxic T cells are sequestered in the lung tissue (45). The host cells undergo persistent apoptosis, necrosis, or pyroptosis, which may amplify the tissue damage. In addition, the inflammatory environment triggers the expression of activated tissue factor on endothelial cells, macrophages, and neutrophils, enhancing activation of the coagulation cascade in the lungs (46). Markers of the coagulation pathway such as upregulation of D-dimer are clearly visible at this stage. In an autopsy study, Wichmann et al. demonstrated that 58% of COVID-19 patients have a concurrency of DVT, and 1/3 of the deaths were directly caused by pulmonary embolism (PE) (47). The potential role of thrombosis in pulmonary veins distal to the alveolar capillary bed, which should act as clot filters, has been pointed out, suggesting that it could be SARS-CoV-2-related vasculitis responsible for ischemic manifestations in various organs (48). For this reason, severe COVID-19 is not restricted to the respiratory system but is a multisystem disease including the development of various cardiovascular manifestations with myocardial injury, arrhythmia, acute coronary syndrome, and venous thromboembolism. These manifestations are closely related to the disease severity and progression to lethal disease (49). Based on all these features, anticoagulant therapy and immunomodulatory agents are probably necessary to attenuate the hyperinflammatory and prothrombotic states (50). These patients may benefit from immune modulators such as steroids while antiviral agents have limited utility at this stage of the disease. Despite the obvious contribution of coagulation pathways in vascular disease, the use of anticoagulant therapy may be filled with the risk of increased bleeding. On the other hand, microcirculatory thrombi in capillaries and large vessels may already cause extensive damage if administered too late.

### 2.4 The complexity of immune response during COVID-19

Exacerbated immune response manifested as cytokine storm is a common pathological event in many infectious diseases. However, given the wide range of clinical presentations and extensive studies performed on COVID-19, a clearer picture of beneficial and pathological immune responses started to emerge. Host immune response, initiated by sensing the pathogen-associated molecular patterns (PAMP) present on the pathogen strives to eliminate the invading pathogen. A multitude of evidence suggests that the initial host response is dampened markedly in severe COVID-19 patients, especially those mediated by type I interferon (51, 52). Type I IFNs (IFN- α, IFN- β, IFN- ω) are indispensable in viral clearance, and this impairment is associated with high blood viral load and exacerbated inflammatory response (33). Additionally, the nucleocapsid (N) protein, one of the four structural proteins of CoV, serves as an antagonist of IFN, which appears to be beneficial for viral replication (53, 54). It was speculated that a large spectrum of severe clinical presentations could result from a delayed host response towards a specific pathogen-associated molecular pattern (PAMP), which attenuates the antiviral innate immunity required to eliminate the pathogen. In support of this hypothesis, treatment with type I interferon has shown beneficial effects in COVID-19 (55).

Moreover, the postponement of the PAMP-mediated response shifts the immune response to a nonspecific inflammatory reaction dominated by damage-associated molecular pattern (DAMP) signaling emanating from damaged or dysfunctional virus-infected host cells (56, 57). Although PAMPs and DAMPs can lead to innate and adaptive immune responses, the latter raises additional damage and dysfunction by releasing various pro-inflammatory cytokines by activating dendritic cells and other antigen-presenting cells (APCs). DAMPs initiate innate immune activation and systemic inflammation, which could further upregulate itself by triggering a cytokine storm, providing positive feedback to tissue destruction. It is not surprising that young subjects who remained asymptomatic post-SARS-CoV-2 infection had elevated levels of inflammatory cytokines such as IL-2 while decreasing levels of the anti-inflammatory cytokine IL-10 (57). The current clinical challenge remains to identify and distinguish the DAMP-driven immune response from that driven by PAMPs.

Similar to the innate immune response, the adaptive immune response to SARS-CoV-2 infection has been studied in detail. Overall, it appears that the ability of the host to generate an early humoral response rather than mounting a stronger humoral response is critical in protecting the host against severe disease. Both delayed onset of the humoral response and elevated antibody titers are associated with severe disease in COVID-19 (58, 59). To support this hypothesis, we have shown that the early onset of antibody response was associated with asymptotic disease and patients with severe disease had elevated antibody response (60, 61). Along similar lines, convalescent plasma therapy provided limited benefits in COVID-19, leading the World Health Organization to issue guidelines against plasma therapy for COVID-19 (62).

### 2.5 Long COVID

Despite the widespread prevalence of long COVID-19, the mechanistic understanding of underlying pathophysiology remains limited. There are several suggested hypotheses for the pathogenesis of long COVID-19 including the persistent exposure of SARS-CoV-2 in tissues, dysregulation of the immune system, reactivation of secondary pathogens (eg. EBV, HHV-6, HCMV, VZV etc.), microbiome dysbiosis in the gastrointestinal system, autoimmunity and immune priming from molecular mimicry, microvascular blood clotting with endothelial abnormalities, and dysfunctional neurological signaling, among others (63–66). Increasing evidence have shown that two third of the population with long COVID is associated with multiple potential risk factors and pre-existing conditions, including female sex, type 2 diabetes, underlying virus reactivation, the presence of specific autoantibodies, and connective tissue disorders (67–69). A higher prevalence of long COVID-19 has been reported in certain ethnicities, including people with Hispanic or Latino heritage (13). Lower income and lack of sufficient rest in the early weeks after SARS-CoV-2 infection are associated with long COVID-19, which is characterized by postexertional malaise, postural orthostatic tachycardia syndrome, pain, fatigue, unrefreshing sleep, brain fog, cognitive dysfunction, gastrointestinal symptoms, neurological symptoms, among others (70, 71).

## 3 Clinical management

### 3.1 Prevention and self-protection

The origin of SARS-CoV-2 including the animal host still needs definitive identification (72). Most of the infections occur through human-to-human contact, either directly through droplets emitted from infected objects or indirectly by aerosols suspended in the air (73). Preventive measures such as mask/respirator use are highly effective if used appropriately, although the quality of the mask/respirator is critically important. Although infections in healthcare workers have been reported, but larger outbreaks in the healthcare workers have been rare, even before the availability of vaccines, due to the efficacy of personal protective equipment. A nationwide cross-sectional study in Bangladesh has proved that adequate preventive health measures were associated with a lower risk of infection and death from COVID-19 (74). Among preventive health measures, washing/cleaning hands with soap or hand sanitizer (OR: 0.17, 95% CI: 0.09–0.41), wearing masks properly (OR: 0.02, 95% CI: 0.01–0.43), avoiding crowded places (OR: 0.07, 95% CI: 0.02–0.19), and maintaining social distancing in public places (OR: 0.04, 95% CI: 0.01–0.33), were significantly associated with the reduced number of cases and deaths (74).

### 3.2 Early isolation and treatment settings

Early isolation and appropriate duration of quarantine are required to stop or slow down the spread of COVID-19 or its newly emerging variants. However, given the ubiquitous spread of COVID-19, the availability of vaccines, effective therapeutic agents, and an overall decrease in the severe disease by new variants, most countries are shifting their focus away from extensive quarantine and isolation of suspected individuals. However, the experience from this pandemic should guide us in future outbreaks to limit both the disease spread and better deal with the social and economic costs associated with the extensive quarantine.

### 3.3 Vaccines

Vaccines are vital cost-effective tools to prevent the disease and limit the disease severity during the COVID-19 pandemic. Effective vaccine plays a critical role in preventing the viral spread and limiting the disease severity (75). Multiple vaccines are currently available including adenovirus vector vaccines, mRNA, inactivated, and subunit vaccines. The initial data shows that among individuals ≥ 18 years of age, adenoviral vector vaccines were 73% (95% CI = 69- 77) effective and messenger RNA (mRNA) vaccines were 85% (95% CI = 82- 88) (76). Existing adenovirus, mRNAs, and inactivated vaccines can elicit significant immune responses against SARS-CoV-2 RBDs in vaccinated recipients, and individuals develop neutralizing antibodies against the specific area within 30 days of the first and second doses of the vaccine (77). However, the efficacy figures of these vaccines are evaluated within the first 6 months post-vaccination. The immunity gradually decreases over time, and breakthrough infections became common. In addition, mutations in the SARS-CoV-2 can subvert the immunity to cause infections in vaccinated subjects. Boosting host immunity with additional doses of vaccines is effective in limiting disease severity and death, especially among older patients. The beneficial effects of boosters among the young subjects are difficult to decipher given low hospitalizations and mortality in these subjects and potentially due to strong immune response to initial vaccine doses. However, in patients with carcinomas and other immunosuppressive diseases, currently available vaccines have shown to trigger sufficient immune response (78, 79). Further, variant-specific vaccines have been recently developed and approved, however, the precise supremacy of variant-specific vaccines in real-life settings is yet to be determined.

### 3.4 Diagnostic evaluation

Reliable clinical or laboratory parameters are of great importance to accurately distinguish between COVID-19 and respiratory infections of other origins. Individuals with typical respiratory symptoms such as fever, cough, and myalgia should be tested by respiratory nucleic acid amplification test (NAAT) through the specimen from bronchoalveolar lavage, sputum, and nasopharyngeal swab using real-time fluorescence polymerase chain reaction with reverse transcription (RT-PCR). Since false-negative results occur in the circumstance of low viral load in the initial screening, repeated testing should be performed if the symptoms continue to persist. Other measures such as computed tomography (CT) examination can be performed for the auxiliary diagnosis of COVID-19 which is manifested as patchy or segmental GGOs (93.3%) and reticular markings distributed by peribronchovascular and subpleural (80). Although limitations exist when some imaging signs of COVID-19 were presented as the same as those of other lung diseases. Chest CT is easy to perform and readily available to quickly detect lung lesions and make imaging diagnoses at an early stage. Particular attention should be paid to the final confirmation of a SARS-CoV-2 infection. In addition, patients with fever in the emergency department (ED) should be monitored for the presence of SARS-CoV-2 (81). While nucleic acid-based tests or antigen detection tests are used for diagnostic purposes, antibody detection tests may be used to assess the overall exposure in the population (82).

### 3.5 Therapy and clinical management

**Figure 2:**
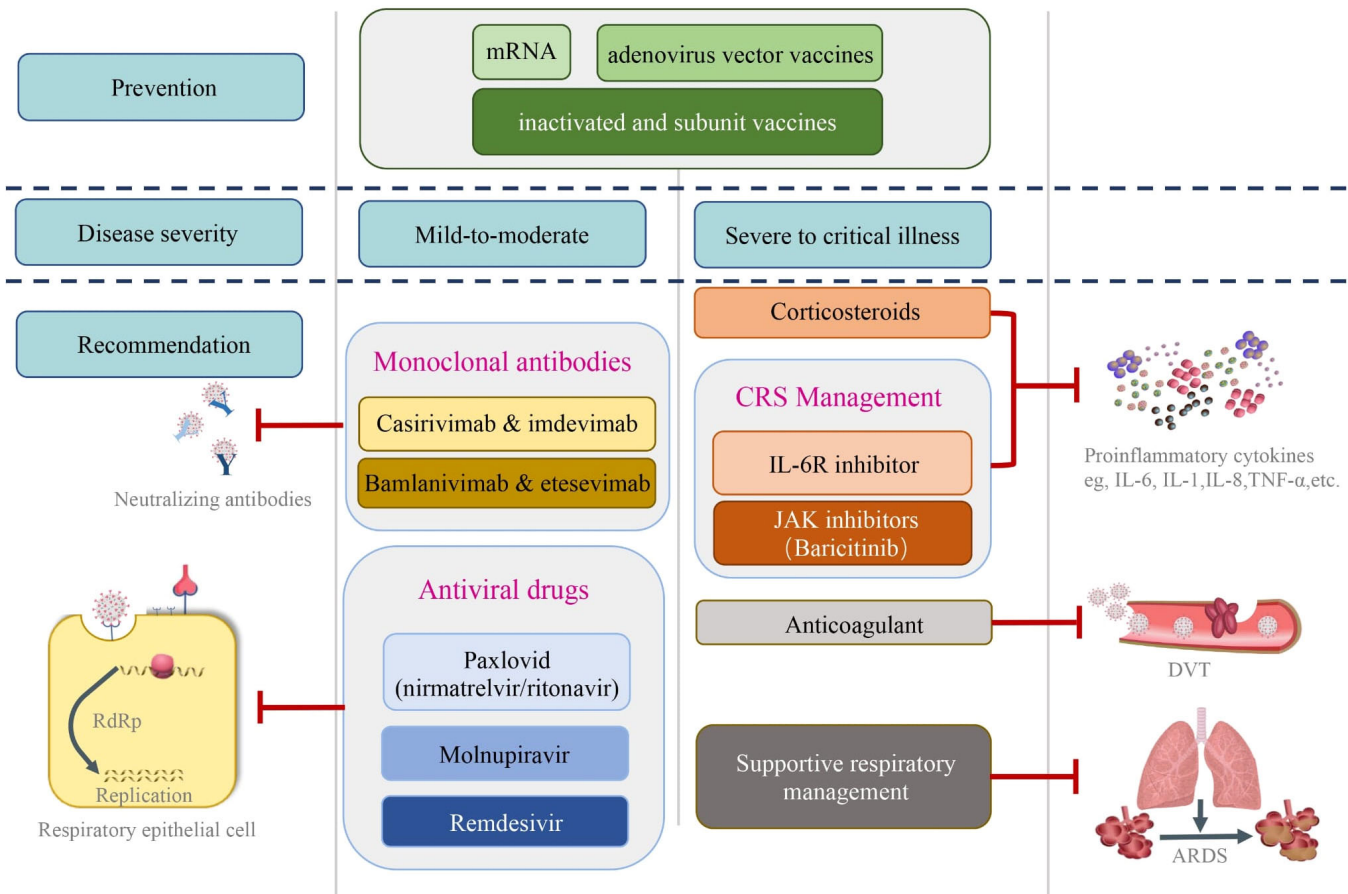
The clinical management and target of currently used therapeutics against SARS-CoV-2. Prior to clinical infection with coronavirus, effective vaccines including adenovirus vector vaccines, mRNA, inactivated, and subunit vaccines play critical roles in preventing virus spread and limiting the severity of the infection. The clinical management and target of currently used therapeutics against SARS-CoV-2 are shown based on the disease severity. Monoclonal antibodies including casirivimab & imdevimab and bamlanivimab & etesevemab have been used in non-severe cases. Likewise, antiviral drugs such as Paxlovid (nirmatrelvir & ritonavir), molnupiravir and remdesivir, which target the virus replication in the infected cells have been used in mild-to-moderate patients. For severe to critically illness, corticosteroids and CRS management including IL-6R inhibitor and JAK inhibitors were administrated to control the excessive inflammation. Anticoagulants have been used to prevent and treat DVT. Supportive respiratory managements have been primary measures to treat patients with low blood oxygenation including ARDS. DVT, deep venous thrombosis; ARDS, acute respiratory distress syndrome.

The clinical management and target of currently used therapeutics against SARS-CoV-2 are demonstrated (**Tables 1**, **2**) (96). The clinical management of COVID-19 involves a wide range of antiviral and immune modulatory drugs that are described below (**Figure 2**).

**Table 1:** Main findings of reported literature.

Authors	Journal	Year of publication	Study type	Main findings
Zhou F et al. (1)	Lancet	2020	Retrospective study	Potential risk factors such as older age and markers of disease severity such as high SOFA score, and elevated d-dimer could identify poor prognosis at an early stage.
Zhu N et al. (2)	N Engl J Med	2019	Descriptive study	Isolation of the virus and designated it as a novel beta coronavirus belonging to the sarbecovirus subgenus of the Coronaviridae family. Further, described its specific cytopathic effects and morphology.
Wiersinga WJ et al. (34)	JAMA	2020	Systemic review	Described detailed aspects of transmission, infection, and treatment.
Spinner CD et al. (83)	JAMA	2020	Randomized Clinical Trial	Investigated the effects of Remdesivir on clinical status at 11 days in moderate COVID-19 patients. Compared with standard care, patients in a 5-day course of remdesivir administration had a statistically significant difference in clinical status, while patients treated in a 10-day regimen of remdesivir did not have a statistically significant difference.
Beigel JH et al. (84)	N Engl J Med	2020	Randomized Clinical Trial	Remdesivir was superior in shortening the time to recovery in adults who were hospitalized with COVID-19.
Wang Y et al. (85)	Lancet	2020	Randomized Clinical Trial	Remdesivir was not associated with statistically significant clinical benefits in adult patients admitted to hospital for severe COVID-19.
Jayk Bernal A et al. (86)	New Eng J Med	2021	Randomized Clinical Trial	Early treatment with molnupiravir reduced the risk of hospitalization or death in at-risk, unvaccinated adults with COVID-19.
Hammond J et al. (87)	N Engl J Med	2022	Randomized Clinical Trial	Treatment of symptomatic COVID-19 with nirmatrelvir plus ritonavir resulted in an 89% reduction in the risk of progression to severe COVID-19 than that with a placebo. No major safety concern was reported.
Dougan M et al. (88)	N Engl J Med	2021	Randomized Clinical Trial	In high-risk outpatients, bamlanivimab plus etesevimab resulted in a lower incidence of COVID-19-related hospitalizations and deaths than placebo and accelerated the decline in SARS-CoV-2 viral load.
Weinreich DM et al. (89)	N Engl J Med	2021	Randomized Clinical Trial	The REGN-COV2 antibody cocktail reduced viral loads, with greater effect in patients whose immune response had not yet started or who had high viral loads at baseline. Safety results were similar in the REGN-COV2 combined dose groups and the placebo group.
Chen P et al. (90)	N Engl J Med	2021	Randomized Clinical Trial	Evaluated the quantitative virologic end points and clinical outcomes in patients receiving a single intravenous infusion of neutralizing antibody LY-CoV555 in one of three doses (700 mg, 2800 mg, or 7000 mg) or placebo. This interim analysis of a phase 2 trial showed that one of three doses (2800 mg) of neutralizing antibody LY-CoV555 appeared to accelerate the natural decline in viral load by day 11.
Cohen MS et al. (91)	JAMA	2021	Randomized Clinical Trial	Among residents and staff in skilled nursing and assisted living facilities, treatment during August-November 2020 with bamlanivimab monotherapy reduced the incidence of COVID-19 infection (8.5% vs 15.2%; odds ratio, 0.43 (95% CI, 0.28-0.68); P < .001; absolute risk difference, -6.6 (95% CI, -10.7 to -2.6) percentage points).
Guimaraes PO, et al. (92)	New Eng J Med	2021	Randomized Clinical Trial	Among patients hospitalized with COVID-19 pneumonia, tofacitinib led to a lower risk of death or respiratory failure on day 28 than placebo (18.1% vs 29.0%; risk ratio, 0.63; 95% confidence interval (CI), 0.41 to 0.97; P = 0.04).
Cao Y et al. (93)	J Allergy Clin Immunol	2020	Randomized Clinical Trial	Ruxolitinib recipients had a numerically faster clinical improvement at day 14 (90% from the ruxolitinib group showed computed tomography improvement compared with 61.9% in control group; P =0.0495). Significant chest computed tomography improvement, a faster recovery from lymphopenia, and favorable side-effect profile in the ruxolitinib group.
Shen C et al. (94)	JAMA	2020	Case series	In this preliminary uncontrolled case series of 5 critically ill patients with COVID-19 and ARDS, administration of convalescent plasma containing neutralizing antibody was followed by improvement in their clinical status with decreasing the SOFA score and increasing the Pao2/Fio2 within 12 days. The limited sample size and study design preclude a definitive statement about the potential effectiveness of this treatment, and these observations require evaluation in clinical trials.
Simonovich VA, et al. (95)	N Engl J Med	2021	Randomized Clinical Trial	No significant differences were observed in clinical status (odds ratio, 0.83; 95% confidence interval (CI), 0.52 to 1.35; P = 0.46) or overall mortality (10.96% in the convalescent plasma group and 11.43% in the placebo group, 95% CI, -7.8 to 6.8) between patients treated with convalescent plasma and those who received placebo.

**Table 2:** Indications and contraindications of drugs in the treatment of COVID-19.

Drugs	Indications	Contraindications
**Antiviral drugs**		
Paxlovid	Paxlovid is a combination therapy for the treatment of mild to moderate COVID-19 in patients who are at high risk of progression to severe COVID-19.	Paxlovid is contraindicated in patients with a known hypersensitivity to any component of the medication.
Molnupiravir	Molnupiravir is an antiviral medication for the treatment of COVID-19 in adults who are at risk of progression to severe COVID-19 and/or hospitalization.	Molnupiravir is contraindicated in patients with a known hypersensitivity to any component of the medication. Also, Molnupiravir is not authorized for use in patients aged <18 years; not authorized for initiation of treatment in patients requiring hospitalization owing to COVID-19; not authorized for use for >5 consecutive days Not authorized for preexposure or postexposure prophylaxis of COVID-19.
Remdesivir	Remdesivir is an antiviral medication for the treatment of COVID-19 in adults and pediatric patients (12 years of age and older and weighing at least 40 kg) requiring hospitalization.	Remdesivir is contraindicated in patients with a known hypersensitivity to any component of the medication.
**Monoclonal antibodies**		
Bamlanivimab and etesevimab	Bamlanivimab and etesevimab are monoclonal antibodies indicated for the treatment of mild to moderate COVID-19 in patients who are at high risk of progression to severe COVID-19 and/or hospitalization.	There are no known contraindications for Bamlanivimab and etesevimab.
Casirivimab and imdevimab	Casirivimab and imdevimab are monoclonal antibodies indicated for the treatment of mild to moderate COVID-19 in patients who are at high risk of progression to severe COVID-19 and/or hospitalization.	There are no known contraindications for Casirivimab and imdevimab.
**IL-6R inhibitor/Immune Modulators**		
Tocilizumab	Tocilizumab is an immunosuppressive medication used to treat moderate to severe rheumatoid arthritis, juvenile idiopathic arthritis, giant cell arteritis, and cytokine release syndrome caused by CAR T-cell therapy or COVID-19.	Tocilizumab is contraindicated in patients with a known hypersensitivity to any component of the medication. For all immunosuppressive treatments, secondary bacterial and fungal infections should be closely monitored.
Sarilumab	Sarilumab is an immunosuppressive medication used to treat moderate to severe rheumatoid arthritis and cytokine release syndrome caused by CAR T-cell therapy or COVID-19.	Sarilumab is contraindicated in patients with a known hypersensitivity to any component of the medication.
Baricitinib	Baricitinib is an immunosuppressive medication used to treat moderate to severe rheumatoid arthritis and COVID-19 in combination with remdesivir.	Baricitinib is contraindicated in patients with a known hypersensitivity to any component of the medication.
Ruxolitinib	Ruxolitinib is an immunosuppressive medication used to treat certain types of bone marrow disorders and cytokine release syndrome caused by CAR T-cell therapy or COVID-19.	Ruxolitinib is contraindicated in patients with a known hypersensitivity to any component of the medication.
Tofacitinib	Tofacitinib is an immunosuppressive medication used to treat moderate to severe rheumatoid arthritis and cytokine release syndrome caused by CAR T-cell therapy or COVID-19.	Tofacitinib is contraindicated in patients with a known hypersensitivity to any component of the medication.
**Systemic corticosteroids**		
Dexamethasone	Dexamethasone is a corticosteroid medication used to treat a variety of inflammatory and autoimmune conditions, as well as to reduce inflammation in certain types of cancer.	Dexamethasone is contraindicated in patients with a known hypersensitivity to any component of the medication. It should also be used with caution in patients with certain infections, such as systemic fungal infections, due to the risk of exacerbating the infection.

#### 3.5.1 Antiviral drugs

In the last four years, aggressive research allowed the development of novel antiviral agents in addition to repurposing other antiviral molecules. These agents vary in efficacy and adverse effect profile. Some of the currently available agents are the following:

##### 3.5.1.1 Remdesivir

Remdesivir was originally developed as an antiviral agent against Ebola, however, the clinical trials failed to show any effectiveness in reducing the mortality (83). The mechanism by which remdesivir acts against viruses is by acting as a nucleoside analog and inhibiting RNA-dependent RNA polymerase, which elicits the delay of the chain termination in the replication of the RNA genome (97). The drug was then tested against SARS-CoV-2, where it was found to have potent antiviral effects during *in vitro* testing (98). These studies led to its approval for emergency use in COVID-19. The initial clinical studies show the clinical benefit of remdesivir in COVID-19 (84). This led to WHO recommending the use of remdesivir for patients without severe or critical illness in April 2022. Subsequent randomized clinical trials failed to show any beneficial effects of remdesivir regardless of the severity of the disease (62, 83, 85). This prompted WHO to recommend against the use of remdesivir in COVID-19 regardless of disease severity (62).

##### 3.5.1.2 Molnupiravir

Molnupiravir is a ribonucleoside prodrug of N-hydroxycytidine (NHC) that effectively inhibits RNA viruses including SARS-CoV-2 (99). Large-scale clinical trials have shown its beneficial effects during COVID-19 including decreasing hospitalizations and mortality (86). Unlike remdesivir, this drug can be used orally, allowing it to be administered early in high-risk subjects. Subsequent studies have confirmed the beneficial findings, but the beneficial effects were limited to a 30% decrease in mortality due to COVID-19 (100, 101). Another major concern is its efficacy against novel variants. Initial data show the clinical benefit of molnupiravir against alpha and beta variants, however, limited clinical data are obtained that molnupiravir exerts activity against delta and omicron variants (86, 102, 103). Recent evidences have indicated the potential benefit of molnupiravir against the omicron variant (104).

##### 3.5.1.3 Paxlovid

Paxlovid is one of the most effective and orally available anticoronaviral medication against SAR-CoV-2. Paxlovid is a combination of nirmatrelvir and ritonavir. Nirmatrelvir targets the main polyprotein protease enzyme of SARS-CoV-2 in the replication cycle, dramatically decreasing the viral loads (105). Ritonavir is a protease inhibitor and a CYP3A4 antagonist, inhibiting nirmatrelvir breakdown and enhancing its pharmacokinetics (106). A double-blind, randomized, controlled trial with 2246 patients who received paxlovid (300mg of nirmatrlvir and 100mg of ritonavir) showed 89% lower incidence of hospitalization and deaths attributed to any cause within 28 days along with decreased viral load (87). Moreover, no obvious safety concerns were observed besides mild generic side effects such as bitter aftertaste, diarrhea, and fatigue. However, one should be careful about drug-drug interactions and should not be co-administrated with other medicines that are metabolized by CYP3A4. Further, this is not indicated in pregnant or breastfeeding patients.

#### 3.5.2 Monoclonal antibodies

Neutralizing antibodies targeting the spike protein of the coronavirus is another important pillar in the fight against SARS-CoV-2 infection, especially in immunocompromised patients (107). Klank et al. reported that monoclonal antibodies (mAbs) such as Bamlanivimab can be used in both preventive and treatment settings (108). In addition, casirivimab and imdevimab also showed a positive protective effects, as SARS-CoV-2 negative individuals who received the specific mAbs appear to have a lower risk of developing COVID-19 after contact with an infected individual (1.5% versus 7.8%; P<0.001). Further, the symptom duration of patients suffering from COVID-19 was shown to be shorter than that of patients on placebo (109). Given these promising protective effects, a combination of bamlanivimab and etesevimab could be a potential treatment option for immunocompromised patients. In a phase 2/3 study, a combination of bamlanivimab and etesevimab was given to patients with malignancies or to those who were in an immunosuppressive status with COVID-19 (88). Compared to the placebo group, patients receiving the drug showed a lower hospitalization rate (2.1% vs. 7.0%; p<0.001) and a significant reduction in viral load at day 7. Other commercially available mAbs such as casirivimab and imdevimab have also shown a positive effect in protecting against reducing viral loads and hospitalization rates and thereby reducing mortality (89, 90). mAbs probably have the greatest beneficial effects in the early phase of coronavirus infection, where viral replication plays an important role (110, 111). Serious adverse effects, including allergic reactions and cardiac issues such as atrial fibrillation, have occurred and require caution following mAbs infusion (91).

In general, monoclonal antibody therapy is recommended for patients with mild to moderate COVID-19 who are at high risk for disease progression. High-risk factors include older age, obesity, diabetes, chronic kidney disease, or immunosuppression. When it comes to the choice of dose, the specific monoclonal antibody and the patient’s weight are taken into consideration. For example, for the monoclonal antibodies bamlanivimab and etesevimab, the recommended dose is 700mg bamlanivimab and 1400mg etesevimab administered together intravenously for patients weighing at least 40 kg (83, 97). The decision to use monoclonal antibodies and the choice of dose should be made by a healthcare professional based on the individual patient’s clinical situation.

#### 3.5.3 Management of cytokine release syndrome

A number of therapies targeting a specific or broad range of cytokines have been tested in COVID-19. Here we describe major approaches to reduce the cytokine storm to alleviate the disease severity.

##### 3.5.3.1 IL-6 receptor blocker

Cytokine storm responses pose a significant risk in infectious diseases including COVID-19. IL-6 is a pleiotropic cytokine stimulating and regulating the immune response during infections. Since IL-6 is identified as the key propagator of the cytokine storm reaction, blocking the two forms of the IL-6 receptor, a membrane-bound and a soluble IL-6 receptor was considered. IL-6 receptor blockers, including tocilizumab and sarilumab, are recommended to be used intravenously for severe or critical illness patients of COVID-19 based on evidence of mortality reduction and decreased requirement of mechanical ventilation (112, 113). However, this is only effective in those with severe disease.

##### 3.5.3.2 Janus (JAK) kinase inhibitors

Janus kinases are important mediators of cytokine storm and inflammation and serve as a potential target for limiting cytokine storm during COVID-19. Baricitinib, ruxolitinib and tofacitinib are three major JAK inhibitors tested for severe or critical COVID-19 patients (92, 93, 114). The three JAK blockers are considered nonspecific despite evident differences. Baricitinib is mainly described as a JAK1/2 inhibitor while ruxolitinib also presents the weak suppression of TYK2 except from JAK1/2, while tofacitinib showed more inhibitory potential on JAK1/3 than JAK2/TYK2 (115, 116). Although large randomized clinical trials have been limited, our meta-analysis demonstrated the overall beneficial effects of JAK inhibitors in COVID-19 (55).

#### 3.5.4 Systemic corticosteroids

Systemic corticosteroids are generally recommended in treating severe and critical COVID-19 patients to control the over-activated inflammatory response despite confounding results (117, 118). Patients younger than 70 years with persistent symptoms for more than seven days and who required mechanical ventilation are shown to benefit from dexamethasone therapy (119). In contrast, no clinical improvement was observed in patients with a shorter duration of symptoms and without supplemental oxygen, even in COVID-19-induced mild to moderate ARDS (120). The optimal timing of the therapy from symptom onset is still a matter of debate. Theoretically, it can be speculated that systemic glucocorticoid use should be avoided until viral replication is under control by the immune system or through effective antivirals. The consensus is arising that corticosteroids should be administered in patients with severe or critical COVID-19, even within seven days of symptoms onset, and non-severe cases should not be treated with corticosteroids even though symptoms occur longer than a week (62).

#### 3.5.5 Convalescent plasma therapy

Convalescent plasma therapy is one of the experimental treatments for SARS-CoV-2 infection. The antiviral neutralizing antibodies collected from the plasma of recovered patients are transfused into the COVID-19 patients with an active infection to enhance the immune response (94, 95, 121). Such plasma therapy has shown a promising recovery rate in H5N1 influenza and Ebola viral disease (122–124). However, due to different methodologies, appropriate antibody titers ranging from 1:100 to 1:2560 have been reported in the existing studies from donor to recipient (125, 126). The range is confusing and the uncertainty of the definitive dose lies in the different limit values (127, 128). WHO living guidelines recommend this therapy applied to patients with severe illness, but only in research settings or clinical trial, for the reasons that convalescent plasma therapy has no significant effect on the indicators such as time to symptom improvement, length of hospital stay, duration of mechanical ventilation, or mortality (62, 129).

#### 3.5.6 Supportive respiratory management

Mechanical ventilation and supplemental oxygen are the primary measures for addressing those who present with low blood oxygenation. High flow nasal cannula (HFNC), Non-invasive ventilation (NIV), and lung-protective invasive mechanical ventilation (IMV) are widely used to support respiration (130, 131). Given the rapid decompensation due to early intubation strategies, HFNC was selected early in the pandemic and used in the populations with hypoxemic respiratory failure and patients often respond well (132, 133). However, HFNC and other NIV devices, including non-invasive bi-level positive pressure ventilation (BPAP) and continuous positive airway pressure (CPAP), may pose a potential risk of aerosolization of the virus. For further safety in HFNC use, evidence has supported the use of a surgical mask (134). CPAP via a mask covering the mouth and nose or helmet NIV is recommended to minimize room air contamination and to better contain aerosol leakage, providing superior oxygenation, pressure, and outcomes (135). In addition, it is recommended that negative pressure rooms be used to reduce the risk of the coronavirus spread in the ambient air (45). A large, international systematic review and meta-analysis was conducted to examine global case fatality rate (CFR) reports in adult patients with COVID-19 who received invasive mechanical ventilation (IMV). The results showed that the overall estimate for the initial CFR in IMV was 45%. Reported CFR was higher in elderly patients and in early pandemic epicenters, which may be impacted by limited ICU resources (136). However, IMV is used for crucial supportive care and to provide additional time for patients with severe hypoxemic respiratory failure in the ICU. Further, invasive mechanical ventilation may increase the risk of secondary bacterial infections in COVID-19 patients by either directly breaching the host defense or through coronavirus-impaired host immunity (137).

## 4 Conclusion

Our understanding of COVID-19 pathology, clinical management, and treatments has improved significantly in the last four years. However, the persistent emergence of variants poses a serious challenge to the effectiveness of both preventive and therapeutic approaches. The clinical management of these infections faces several problems, including failure to administer early antiviral agents, high false-negative diagnosis rates, mixed reports on certain therapeutic drug efficacy, and rapid progression to severe conditions such as ARDS, pulmonary embolism, disseminated intravascular coagulation, sepsis, and cytokine storm. The global fight against COVID-19 is likely to continue for a long time until we develop effective and clinically proven antiviral therapeutics or vaccines that completely prevent transmission of the disease. Further, as we emerge from the pandemic, special emphasis should be given to the chronic consequences such as long COVID-19, which may become a serious healthcare challenge in upcoming years.

## Author contributions

YZ wrote the initial draft, prepared the figures, and tables. LS and DC edited the manuscript and provided guidance. All authors contributed to the article and approved the submitted version.
